# Ankle sprain as a work-related accident: status of proprioception after 2 weeks

**DOI:** 10.7717/peerj.4163

**Published:** 2017-12-15

**Authors:** Salvador González-Iñigo, Pedro V. Munuera-Martínez, Guillermo Lafuente-Sotillos, José M. Castillo-López, Javier Ramos-Ortega, Gabriel Domínguez-Maldonado

**Affiliations:** 1 ASEPEYO, Seville, España; 2 Department of Podiatry, University of Seville, Seville, Spain

**Keywords:** Ankle sprain, Proprioception, Workplace, Labor consequences

## Abstract

**Purpose:**

This study aims at verifying whether proprioception is abnormal or not, two weeks after a grade 1 and 2 ankle sprain in the scope of work-related accident.

**Methods:**

A descriptive, observation and transversal study was designed to compare speed, movement and oscilation of centre of pressure in employees of companies signed up to a mutual company. Participants’ healthy feet comprised the control group, and feet that had undergone an ankle sprain due to a work-related accident comprised the cases group. The following stability tests were undertaken to both the healthy and injuried feet using a force plate: Monopodal Romberg test with eyes open, Monopodal Romberg test with eyes open on a 30 mm thick foam rubber, Monopodal Romberg test with eyes closed, and Romberg test as monopodal support with eyes closed on a 30 mm thick foam rubber. A multiple logistic regression analysis was performed. From the results of this regression model the COR curve test was performed.

**Results:**

71.7% accuracy in the predictions was attained. The equation was as follows: *Condition (injured or healthy)* = *0.052*⋅% *RGC AP Movement − 0.81*⋅*MREO AP Movement*. The variable MREO antero-posterior movement was used in the COR curve methodology. The area under the curve was greater than 0.65 and at a 95% confidence interval the 0.75 value was included, which in our case was the injured subject condition. Values for sensitivity, specificity, positive predictive value and negative predictive value were 0.667, 0.633, 64.5%, and 65.5%, respectively.

**Conclusion:**

The participants in this study showed a diminished capacity for postural control in an ankle two weeks after an ankle sprain.

## Introduction

In everyday activities we depend on signals coming from our moving bodies to be able to respond to the space around us and react rapidly in changing circumstances. This knowledge about position and movement of the limbs is known as proprioception, and is provided by sensations arising in proprioceptors (Proske & Gandevia, 2012). The postural control depends on visual, vestibular and proprioceptive information (Proske & Gandevia, 2012; Peydro De Moya, Baydal Bertomeu & Vivas Broseta, 2005). Ankle sprain may affect postural control due to proprioceptors injury (Akbari et al., 2006).

Ankle sprain (AS) is the second most common work-related pathology after lower back pain in Spain, with approximately 9% to 12% of leave taken because of temporary work-related incapacity (Juan De Felipe, Velasco & Maestro, 2009; Sáinz De Murieta et al., 2005). This leave usually lasts 20 days on average. Sprains are treated in accordance with the symptoms, and subjects return to work once they remit.

Sprain occurs accidentally and injures the ankle ligaments, which play an important role in proprioception. The most commonly involved are the external ligaments (Otter, 1999; Akbari et al., 2006; Dodangeh-Gonzalez et al., 2013), by means of a sudden foot inversion movement. When a sprain occurs, there is inflammation, ecchymosis, pain and functional alterations because of ligaments and other surrounding tissue distension. The involvement of proprioceptive pathways that occurs favours functional ankle instability (FAI), and over time even chronic ankle instability (CAI). Proprioception, the vestibular and the visual systems are responsible for maintaining balance (Leanderson & Wykman, 1993; Jeka & Lackner, 1994; Slobounov et al., 2005). The information provided by these three systems is integrated by the central nervous system (CNS) to regulate balance and posture. Recovering proprioception after AS is essential to avoid new sprains and for the patient to feel safer.

For grade 1 and 2 AS, objective clinical recovery is quite fast, and patients may present minimal symptoms two weeks from the AS. This situation favours return to work without knowing the person’s proprioception status. If CAI develops, this could cause suffering and impaired articular cartilage with the possibility of developing osteochondritis and subsequently severe arthrosis. In some cases, this process generates a highly debilitating situation for the patient at work with medical and economic impact and significant limitation to daily life activities.

Most research on this regard is focused on sportspeople (Akbari et al., 2006; Leanderson & Wykman, 1993; Evans, Hertel & Sebastianelli, 2004; Aydin et al., 2002; Verhagen et al., 2005; Docherty, Valovich McLeod & Shultz, 2006) or elderly people (Schieppati et al., 1994; Black, 2001; Alexander et al., 1996; Laudner & Koschnitzky, 2010; Martín Nogueras, 2007). To the best of our knowledge, there are no studies that have evaluated proprioception after an ankle strain in the scope of work-related accident. This study aims at verifying whether proprioception is abnormal two weeks after a grade 1 and 2 ankle sprain in the scope of work-related accident.

## Material and Method

### Study design

A descriptive, observational and transversal study was designed to study the differences in behaviour of the centre of pressure (CoP) and proprioception of healthy feet and feet that have undergone an ankle sprain, two weeks after this occurred. The period of 2 weeks was similar to that used in other studies (Evans, Hertel & Sebastianelli, 2004; Doherty et al., 2014; Leanderson et al., 1999; McKeon & Hertel, 2008a; Hertel, Buckley & Denegar, 2001). The present study has been carried out following the STROBE criteria.

### Participants

Participants were employees of companies signed up to the mutual company XXXX who, due to a work-related accident, attended this mutual seeking medical treatment in Seville between February 2012 and June 2014. Therefore, they had to comply with the inclusion criteria and voluntarily agree to be included in the study. All participants gave written consent. The Experimentation Ethics Committee of the University of Seville approved the study.

Inclusion criteria were: being an active employee of working age (in Spain, aged between 18 and 65); having a medical diagnosis of grade 1 or 2 ankle sprain in just one ankle (Mann et al., 2002; Fong et al., 2009), of less than 2 weeks clinical course; not having undergone a sprain in the contralateral ankle or knees in the last 12 months; not suffering from disorders related to the vestibular system; not having undergone osteoarticular surgery of the ankle or subtalar joint or significant trauma that could have modified normal anatomy in these joints, or altered the sensory and motor pathways (Michelson & Hutchins, 1995); not presenting degenerative osteoarticular diseases and/or neuromuscular imbalances; and not taking sedative treatments of the vestibular system, antidepressants, anxiolytics or similar substances that delay or impair the sensory and motor system’s speed of response.

### Procedure

The evaluation protocol was performed using a force plate Dinascan/IBV^®^ (Biomechanical Institute of Valencia, Valencia, Spain). Five tests were undertaken, one control and four effective, according to the protocol set out by the Biomechanical Institute of Valencia (BIV) (Asociación Instituo de Biomecánica de Valencia, 2017). The force plate had an active area of 600 × 370 mm, 100 mm height, 25 kg weight and a measurement range of vertical and horizontal strength of 4,500 N and + 750 N, respectively. It had a sampling frequency of up to 1,000 Hz. The force plate was embedded in a floorboard with a mechanical structure to house the platform measuring 3.5 × 1.5 m. The application NedRodilla/IBV^®^ (Biomechanical Institute of Valencia, Valencia, Spain) was used to analyse leg stability data form platform.

First, the bipodal Romberg’s test was performed on a 90 mm foam rubber with the eyes closed (BREC90 mm) to elucidate whether or not the participant had altered the vestibular system. If a positive outcome was obtained, the participant was excluded from the study. To perform this test a foam rubber 90 mm thick, 56.7 kg/m^3^ density and penetration resistance at 25% of 246 N, was used (Asociación Instituo de Biomecánica de Valencia, 2017; Baydal-Bertomeu et al., 2004). Once the foam rubber was placed on the force plate, the participant was asked to stood on it barefoot with arms extended along the body, looking straight ahead, focused on a fixed far away point and as quiet as possible. They were asked to close their eyes and then the 30-second test began. This was repeated three times.

**Figure 1:**
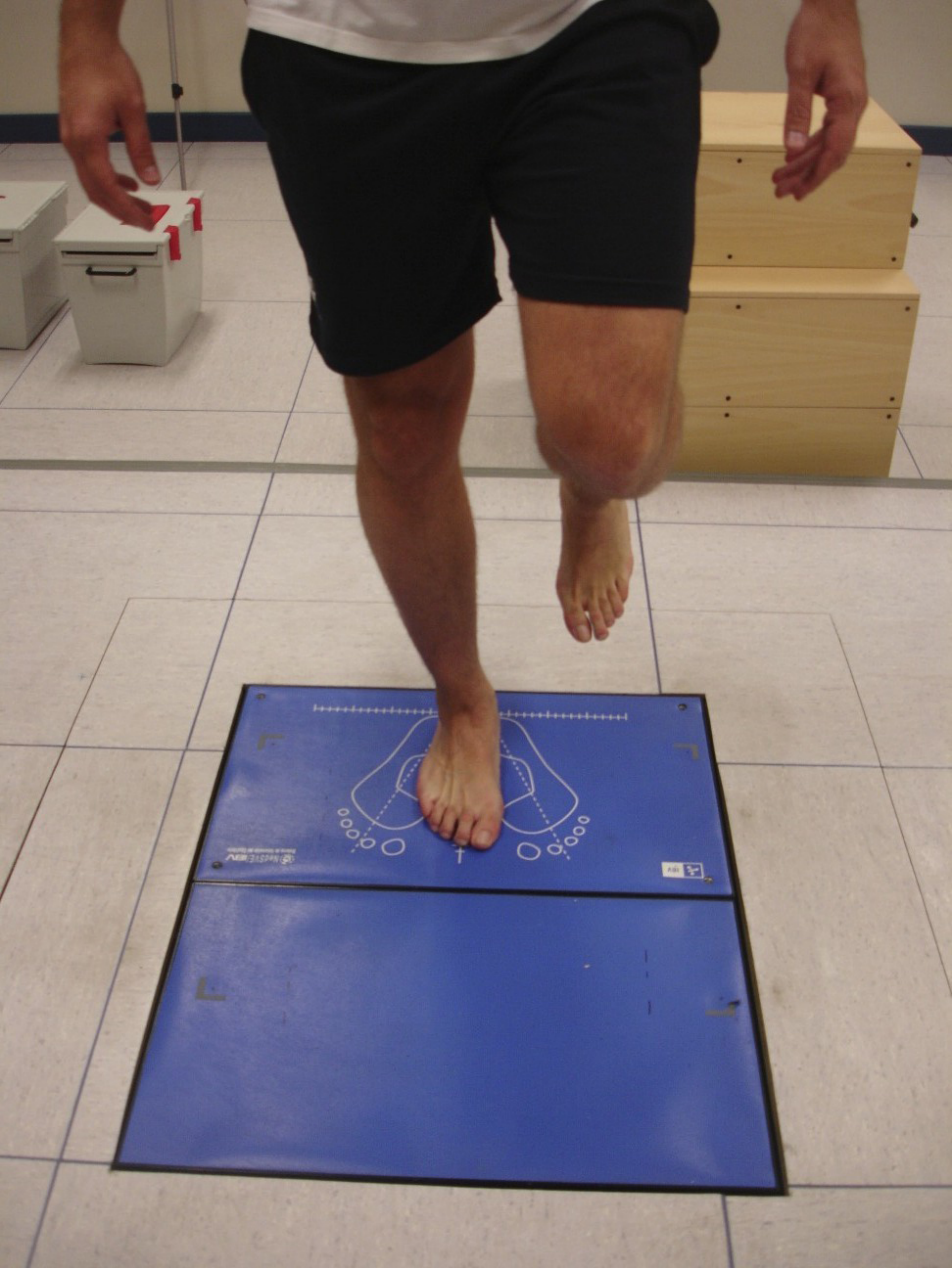
Monopodal Romberg’s test with eyes open.

The following tests were then performed:

– Unipedal Romberg’s test with eyes open (MREO): the participant was positioned with unipdeal weight bearing with the contralateral foot placed at half height the supporting leg (Fig. 1).
– Unipedal Romberg’s test with eyes open on a 30 mm thick foam rubber (MREO30mm): the participant stood in the same way as MREO, but on the force plate there was a 30 mm thick foam rubber, with the same size and shape as the platform.
– Unipedal Romberg’s test with eyes closed (MREC): same test as the MREO but with the eyes closed.
– Romberg’s test as unipedal weight bearing with eyes closed on a 30 mm thick foam rubber (MREC30 mm): same test as the MREO30 mm but with the eyes closed.

The test always began with the right foot and alternated with the left foot (as manufactured suggest), until three measurements were taken for each foot to use the mean of the three measurements during the statistical analysis. Participants had to look straight ahead at a fixed point and they could not speak for the 30 s each measurement lasted. The arms had to remain extended over the body without removing them to keep balanced and avoid contact of one leg with the other.

### Measurements

Measurements were taken by means of the participants’ record of variations in CoP when they were on the force plate. The following records were obtained:

1. Area swept (mm^2^): this is the area swept by the CoP during the test.
2. 75th percentile speed (mm/s): determines the 75th speed percentile for foot correction to maintain balance.
3. Mediolateral movement (mm): evaluation of movement of the participant’s CoP along the mediolateral axis.
4. Anteroposterior movement (mm): evaluation of movement of the participant’s CoP along the anteroposterior axis.
5. Frequency of mediolateral oscillation (Hz): evaluates the characteristic frequency of the participant’s statokinesiogram signal along the mediolateral axis.
6. Frequency of anteroposterior oscillation (Hz): evaluates the characteristic frequency of the participant’s statokinesiogram signal along the anteroposterior axis.
7. Test time (%): represents the percentage total test duration time (15 s) that the participant was able to maintain balance without touching the floor with their contralateral foot.
8. MREO evaluation (%): determines a relative value obtained from the mean percentage values for all variables obtained during the tests performed with eyes open.
9. MREO30mm evaluation (%): determines a relative value obtained from the mean percentage values for all variables obtained during the tests performed on foam rubber with eyes open.
10. MREC evaluation (%): determines a relative value obtained from the mean percentage values for all variables obtained during the tests performed with eyes closed.
11. MREC30 mm evaluation (%): determines a relative value obtained from the mean percentage values for all variables obtained during the tests performed with foam rubber with eyes closed.
12. Final eyes open evaluation (EO) (%): determines a relative value obtained from the mean values of the variables “MREO Evaluation” and “MREO30mm Evaluation”.
13. Final eyes closed evaluation (EC) (%): determines a relative value obtained from the mean values of the variables “MREC Evaluation” and “MREC30 mm Evaluation”

During the four measurements, the value increases with balance disorders, age and difficulty of the test and in oscillation frequency tests the value reduces with balance disorders, age and test difficulty.

Measurements 1 to 6 were also expressed as a percentage after comparing the absolute value obtained with a gold standard database from healthy people (Baydal-Bertomeu et al., 2004). The database was validated by the BIV for all their equipment (Asociación Instituo de Biomecánica de Valencia, 2017). For all tests expressed as a percentage, the value decreases with balance disorders.

### Sample size calculation

To calculate the sample size in this study, the overall final valuation of the tests with eyes open (EO)(%) was taken as a reference variable because this is a variable that all participants managed to complete because it was less demanding. Therefore, it was desirable that at least in the eyes open test healthy and injured individuals obtained a 95% and 65% score, respectively. This means it was being implicitly determined that the difference in minimal proportions that we wished to detect is worth the difference between both.

\begin{eqnarray*}& & {P}_{\mathrm{Healthy}}=0.95 \end{eqnarray*}

\begin{eqnarray*}& & {P}_{\mathrm{Injured}}=0.65 \end{eqnarray*}

\begin{eqnarray*}& & D={P}_{\mathrm{Healthy}}-{P}_{\mathrm{Injured}}=0.3. \end{eqnarray*}

The mean proportion was calculated, *P*_*M*_

The risk *α* was set at 5% bilateral, whereby *Z*_*α*∕2_ is equal to 1.96.

\begin{eqnarray*}& & \alpha /2=0.025 \end{eqnarray*}

\begin{eqnarray*}& & {Z}_{\alpha /2}=1.96. \end{eqnarray*}

The risk *β* was determined to be 20%. Therefore, the power was 80% (1-*β*).

\begin{eqnarray*}& & \beta =0.2 \end{eqnarray*}

\begin{eqnarray*}& & {Z}_{\beta }=0.84. \end{eqnarray*}

The equation to apply for the calculation of sample size for each group was:

\begin{eqnarray*}n= \frac{2\cdot {p}_{M}\cdot {q}_{M}\cdot ({z}_{ \frac{\alpha }{2} }+{z}_{\beta })^{2}}{({p}_{\text{healthy}-}{p}_{\text{affected}})^{2}} = \frac{2\cdot 0.80\cdot 0.2\cdot (1.96+0.84)^{2}}{(0.95-0.65)^{2}} =27.88. \end{eqnarray*}

The calculation of sample size revealed that 28 feet were necessary in each group. Finally, 30 feet were included in each group.

### Data analysis

Data were analysed with the software IBM SPSS Statistics 22^®^ (IBM, Armonk, NY, USA). Normality (Shapiro–Wilk) and randomness tests were performed to determine the use of parametric or nonparametric tests. Parametric tests (*t*-test for equality of means) were used when the data followed a normal distribution and they were random. Otherwise, the Mann–Whitney U test was used for independent samples. To ascertain the predictive level that could be attained from the condition of an ankle (injured or healthy), regression analysis was applied based on variables that had statistically significant differences. Subsequently, a multiple logistic regression model was performed for all variables that presented statistically significant differences. From the results of this regression model the COR curve test was performed to find a predictive cut-off point, and positive and negative predictive values were calculated.

## Results

The overall sample of this study was comprised by 30 people (30 left ankles and 30 right ankles), of which 13 were women and 17 were men, with a mean age of 34.43 + 7.28 years. BMI was 26.15 ± 4.72. The 30 subjects presented 22 grade 1, and eight grade 2 strains in one of their ankles. The experimental group was comprised of 30 injured ankles. The control group was comprised of 30 contralateral healthy ankles.

Table 1 shows the descriptive results of all the variables. Table 2 shows the variables that revealed statistically significant differences when compared between the case study group and the control group.

A regression analysis was carried out to predict the participant’s condition (injured or healthy). Considering that most variables that revealed statistically significant differences between both groups were eyes closed tests (among them the overall variable EC evaluation) it was decided to use this variable independently in the logistic regression applied (Table 3). The resulting equation was:

\begin{eqnarray*}\text{Condition}=-3.426+0.053\cdot \text{EC evaluation}. \end{eqnarray*}

As the constant and EC evaluation obtained a value *P* < 0.05, and the Odds Ratio was statistically significant, the logistic equation that enables calculating the probability of being injured or healthy, according to the overall evaluation of eyes closed tests was as follows:

If the probability is higher than 0.5 the prediction would be injured. If it is lower, the prediction would be healthy. If what was revealed in this analysis had been applied, it would have obtained a participant classification as shown in Table 4.

Using the data from Table 4 and with a risk *α* = 0.05 it can be stated that of a total of 60 individuals, 65% were correctly classified as injured or healthy. To evaluate the predictive capacity of the model, sensitivity (56.7%) and specificity (73.3%) values were calculated. These values were acceptable and correct, respectively.

A multiple logistic regression analysis was performed with all variables that were previously statistically significant (those that appear in Table 2). The backward steps method was used and the final model was reached in step number 11 (Tables 5 and 6). As can be seen in Table 5, 71.7% accuracy in the predictions was attained. The equation was as follows:

\begin{eqnarray*}\text{Condition (injured or healthy)}& =& 0.052\cdot \text{% RGC AP Movement}\nonumber\\\displaystyle & & -\,0.81\cdot \text{MREO AP Movement}. \end{eqnarray*}

When both variables led to statistical significance variables less than 0.05, it made sense to use them in this predictive model.

**Table 1:** Mean ± Standard deviation from the measurements recorded.

	Final evaluation EO/EC (%)		Evaluation (%)		Test time (%)		ML movement (mm)		ML movement (%)		AP movement (mm)		AP movement (%)	
	E	S	E	S	E	S	E	S	E	S	E	S	E	S
MREO	97.0 ± 4.0	98.2 ± 2.2	97.0 ± 6.0	98.2 ± 2.0	100.0 ± 0.0	100.0 ± 0.0	30.5 ± 14.6	25.5 ± 5.4	97.0 ± 9.0	99.7 ± 1.0	39.8 ± 14.2	34.6 ± 6.4	96.8 ± 8.0	99.4 ± 1.0
MREO30mm			98.2 ± 3.0	98.4 ± 2.0	100.0 ± 0.0	100.0 ± 0.0	26.8 ± 4.2	26.9 ± 5.5	99.9 ± 1.0	99.8 ± 1.0	36.8 ± 7.6	33.8 ± 6.9	99.1 ± 2.0	99.7 ± 1.0
MREC	59.4 ± 16.3	69.6 ± 12.0	66.6 ± 20.8	77.1 ± 12.9	78.9 ± 22.7	90.2 ± 13.0	45.5 ± 14.1	45.2 ± 7.0	75.2 ± 25.0	86.6 ± 14.0	68.1 ± 14.9	69.7 ± 11.1	67.1 ± 22.0	78.4 ± 13.6
MREC30mm			52.9 ± 16.2	62.8 ± 14.7	65.1 ± 20.4	76.8 ± 20.2	54.6 ± 23.5	52.2 ± 20.8	57.2 ± 21.0	65.7 ± 17.0	75.9 ± 8.4	74.8 ± 13.9	51.4 ± 17.1	62.3 ± 15.6

	Area swept (mm^2^)		Area swept (%)		75th percentile speed (mm/s)		75th percentile speed (%)		Frequency of ML Oscillation (Hz)		Frequency of ML Oscillation (%)		Frequency of AP Oscillation (Hz)		Frequency of AP Oscillation (%)	
	E	S	E	S	E	S	E	S	E	S	E	S	E	S	E	S
MREO	1,251.2 ± 1,749.7	695.9 ± 257.9	94.4 ± 18.1	97.3 ± 7.0	68.9 ± 13.4	66.1 ± 14.6	99.0 ± 2.0	99.3 ± 2.0	5.1 ± 1.0	5.3 ± 0.8	96.0 ± 4.9	97.6 ± 3.8	3.4 ± 0.7	3.4 ± 0.8	96.0 ± 5.0	95.6 ± 8.0
MREO30mm	774.6 ± 323.5	730.9 ± 286.9	95.1 ± 12.0	96.6 ± 8.0	68.4 ± 11.5	67.2 ± 15.0	99.5 ± 2.0	99.3 ± 3.0	5.7 ± 0.8	5.2 ± 0.9	97.5 ± 5.0	97.5 ± 4.0	3.7 ± 0.7	3.5 ± 0.9	97.6 ± 5.0	97.1 ± 6.3
MREC	2,981.6 ± 1,283.7	2,881.3 ± 100.4	36.8 ± 20.1	42.9 ± 20.9	120.8 ± 21.4	127.4 ± 23.0	63.4 ± 21.2	73.2 ± 16.1	5.4 ± 2.4	4.8 ± 0.7	76.9 ± 24.8	90.8 ± 13.3	4.7 ± 1.1	4.5 ± 0.7	80.6 ± 25.0	91.5 ± 13.0
MREC30mm	4,409.2 ± 271.5	3,660.9 ± 1,423.2	24.6 ± 11.3	31.5 ± 12.2	131.1 ± 17.2	130.8 ± 21.1	50.5 ± 16.8	59.8 ± 17,9	5.4 ± 1.7	4.8 ± 0.8	65.4 ± 23.0	79.5 ± 20.0	4.9 ± 1.2	4.3 ± 0.8	66.6 ± 19.4	78.3 ± 19.1

**Notes.**

AP Antero-posterior H Healthy ML Medial-lateral S Sprain

**Table 2:** Summary of the results of comparisons between the sprains and control groups, and of those variables that revealed statistically significant differences with the Student *t* test for independent samples (*) and the Mann–Whitney *U* test.

Variables with significant results	Mean ± Standard deviation Injured	Mean ± Standard deviation Healthy	P
MREC 75th percentile speed (%)	63.4 ± 21.2	73.2 ± 16.1	0.048 ^∗^
Variation MREC30mm	52.9 ± 16.2	62.8 ± 14.7	0.016 ^∗^
MREC30mm AP movement (%)	51.4 ± 17.1	62.3 ± 15.6	0.013 ^∗^
MREC30mm 75th Percentile speed (%)	50.5 ± 16.8	59.8 ± 17.9	0.042 ^∗^
EC evaluation	59.4 ± 16.3	69.6 ± 12.0	0.008 ^∗^
MREC30mm Frequency of AP Oscillation (Hz)	4.9 ± 1.2	4.3 ± 0.8	0.038 ^∗^
MREO AP movement (mm)	39.8 ± 14.2	34.6 ± 6.4	0.037
MREC test time (%)	78.9 ± 22.7	90.2 ± 13.0	0.033
MREC Frequency of ML Oscillation (%)	76.9 ± 24.8	90.8 ± 13.3	0.011
MREC30mm test time (%)	65.1 ± 20.4	76.8 ± 20.2	0.027
MREC30mm Area swept (%)	24.6 ± 11.3	31.5 ± 12.2	0.013
MREC30mm Frequency of ML Oscillation (%)	65.4 ± 23.0	79.5 ± 20.0	0.012
MREC30mm Frequency of AP Oscillation (%)	66.6 ± 19.4%	78.3 ± 19.1	0.015

The variable AP movement for both tests was a good predictor to ascertain whether or not a participant was injured.

**Figure 2:**
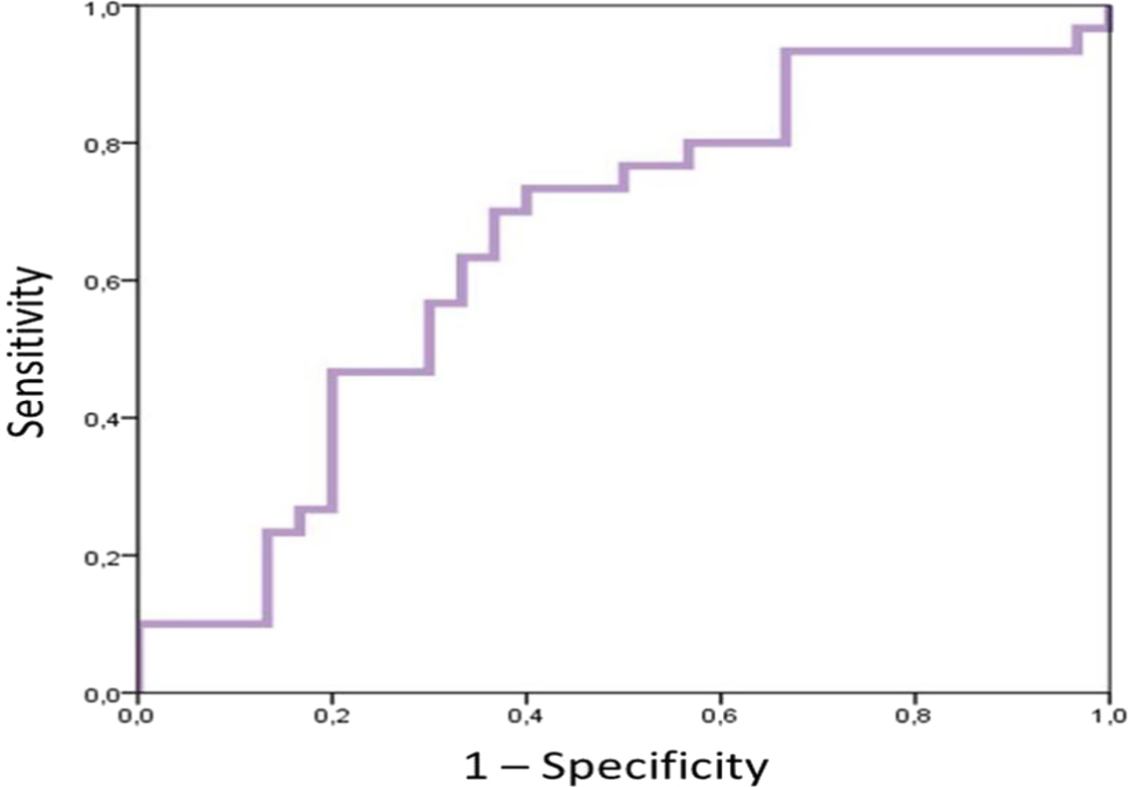
COR graphic.

From the results of this regression model the COR curve test was performed to find a predictive cut-off point, and positive and negative predictive values were calculated. The variable “MREO AP Movement” was used, because all eyes open tests were fully performed by injured and healthy subjects and because it was more important in the equation because its coefficient had a higher value than the “% MREC30mm AP Movement”. The area under the curve (AUC) was greater than 0.65 and at a 95% confidence interval the 0.75 value was included, which was the injured participant condition (Fig. 2).

**Table 3:** Variables from the equation in the regression model.

Variables in the equation									
		B	Standard error	Wald	gL	Sig.	Exp(B)	95% CI for EXP(B)	
								Inferior	Superior
Step 1	EC evaluation	0.053	0.021	6.062	1	0.014	1.054	1.011	1.099
	Constant	−3.426	1.432	5.722	1	0.017	0.033		

**Notes.**

Variables specified in step 1: EC evaluation.

**Table 4:** Classification table.

Classification table^a^					
			Predicted		
			Condition		Percentage correction
	Observed		Injured	Healthy	
Step 1	Condition	Injured	17	13	56.7
		Healthy	8	22	73.3
	Overall percentage				65.0

**Notes.**

a The cut-off value is 0.5.

**Table 5:** Classification table (Step 11).

Classification table^a^					
			Predicted		
			Condition		Percentage correction
	Observed		Injured	Healthy	
Step 11	Condition	Injured	20	10	66.7
		Healthy	7	23	76.7
	Overall percentage				71.7

**Notes.**

a The cut-off value is 0.5.

**Table 6:** Variables in the equation (Step 11).

Variables in the equation									
		B	Standard error	Wald	gL	Sig.	Exp(B)	95% CI for EXP(B)	
								Inferior	Superior
Step 11	% MREC30mm AP movement	0.052	0.018	8.765	1	0.003	1.054	1.018	1.091
	MREO AP movement	−0.081	0.028	8.287	1	0.004	0.922	0.872	0.974

The AUC in the sensitivity column was taken as optimal value and the sensitivity, specificity, positive predictive value and negative predictive value were 0.667, 0.633, 64.5% and 65.5%, respectively. According to these results the value to predict whether or not there was an injury was that the variable “MREO AP Movement” was greater than 35.4875. According to this criterion in this sample results would be as shown in Table 7.

**Table 7:** Condition with MREO AP movement.

	Less than or equal to 35.4875	Greater than 35.4875	Total
	*N*	*N*	*N*
Number healthy	19 (True Negatives)	11 (False Positives)	30
Number injured	10 (False Negatives)	20 (True Positives)	30

## Discussion

The main aim of this study was to determine whether proprioception was altered two weeks from the ankle strain occurring. The results suggest the importance of the relative variables (percentage) of the tests performed with eyes closed, and especially on the rubber foam to confirm that proprioception was altered in the measurements performed at two weeks.

The postural control depends on visual, vestibular and proprioceptive information (Proske & Gandevia, 2012; Peydro De Moya, Baydal Bertomeu & Vivas Broseta, 2005). The latter can be evaluated through the Romberg test in its multiple variants, which is used as a proprioception assessment tool in clinical neurology (García-Pastor & Álvarez Solís, 2014). The fact of studying both ankles (healthy and injured) allows us to isolate proprioception by sharing the same vestibular and visual system in the same circumstances. Stabilometry analyses postural control in stable standing and in conditions of destabilization, by recording the oscillations of the centre of gravity (its projection to the ground) with a force plate.

Ankle sprain is an injury that causes increased postural swing (Akbari et al., 2006; Evans, Hertel & Sebastianelli, 2004; Leanderson et al., 1999; Michelson & Hutchins, 1995; Goldie, Evans & Bach, 1994; Lentell et al., 1995; Bullock-Saxton, Janda & Bullock, 1994) due to involvement of the mechanoreceptors responsible for proprioception (Michelson & Hutchins, 1995), and due to involvement of one of the joints responsible for carrying out strategies to maintain adequate postural control together with the hip (Baydal-Bertomeu et al., 2004; Günther et al., 2009). These mechanoreceptors are injured during the mechanism of production of the sprain (Michelson & Hutchins, 1995), and the subsequent inflammatory process (Evans, Hertel & Sebastianelli, 2004), generating a deficit of transmission of the information that normally occurs.

In the references revised in this study the authors found varying methodologies to perform the unipedal test both in regard to duration, positioning of the subject, repetitions and test conditions (Leanderson & Wykman, 1993; Aydin et al., 2002; Docherty, Valovich McLeod & Shultz, 2006; Doherty et al., 2014; McKeon & Hertel, 2008b; Lysholm et al., 1998; Eils & Rosenbaum, 2001; Riemann et al., 2004; Mitchell et al., 2008b; Mitchell et al., 2008a). We believe that the position of the arms alongside the body as shown by the manual of NedRodilla/IBV^®^ (Asociación Instituo de Biomecánica de Valencia, 2017) is the best option because it does not allow waist movement to compensate the imbalance. By having to perform the test with eyes closed, the duration should be no longer than 15 s, considering that eyes open and closed conditions and a rubber foam to hinder proprioception will be used. In this study, few individuals completed the 15 s during the tests without vision, even with the healthy foot. Regarding test conditions, introducing the eyes closed condition involves that two systems control balance, vestibular and proprioceptive; but if rubber foam is also involved, proprioception has to work better. Among the 13 variables with statistically significant differences observed, 11 were relative variables (%) obtained from comparison of absolute variables with the healthy population database (Asociación Instituo de Biomecánica de Valencia, 2017). The fact that statistically significant differences were not obtained in absolute variables and found just the opposite for relative value variables, could be accounted for by incapacity to finish the test when its difficulty increases. Not completing the test entailed not accumulating enough movement, area, etc. as to generate a statistically significant difference with the healthy foot during the 15 s, as was expected, because it is an injured limb. The values were even lower for the injured ankle compared to the healthy ankle.

The results of this work reveal that variables that presented statistically significant differences were those resulting from the Romberg’s test with eyes closed (except AP movement during MREO). This lends importance to these tests to improve the ability of detection of mild proprioception deficits which could pass unnoticed with eyes open tests. These findings contrast with those of Lysholm et al. (1998) who compared data between injured and healthy legs from participants with AS and found that there were statistically significant differences in the eyes open test, but not the eyes closed test. There is also agreement with Bernier, Perrin & Rijke (1997), who studied participants with FAI and did not find statistically significant differences when performing static and dynamic tests in unipedal weight bearing with eyes open and eyes closed. Conversely, Akbari et al. (2006) found greater differences in the eyes open tests and Mitchell et al. (2008b) did not obtain statistically significant differences in the eyes open test, although they did obtain greater values for movements of injured subjects and statistically significant differences in the eyes closed tests. This finding could suggest that the integrity of two of the three systems for balance control would compensate the deficit of the remainder (Akbari et al., 2006; Nashner, 1971; Diener & Dichgans, 1988; Horak, Nashner & Diener, 1990; García-Pastor & Álvarez Solís, 2014), which could go unnoticed under eyes open conditions. However, by removing vision, balance control is achieved by the vestibular and proprioceptive system, that are not entirely functional. The afferent signals that arrive are severely reduced and balance control is defective.

Romberg’s test with eyes open did not present statistically significant differences (except AP movement during MREO), although there was a trend for values in the AS group to be greater than the healthy group as observed by other authors (Bernier, Perrin & Rijke, 1997; Ross & Guskiewicz, 2003; Isakov & Mizrahi, 1997). The difference in value in the Romberg’s tests with eyes open may have not been so obvious because of the fact that the systems not affected (vestibular and visual) compensated the proprioception deficit (Akbari et al., 2006); it could also be due to a bilateral proprioception abnormality in people with AS because of abnormal central control mechanisms (Evans, Hertel & Sebastianelli, 2004; Leanderson et al., 1999; Lysholm et al., 1998; Isakov & Mizrahi, 1997; Bastien et al., 2014). Of the Romberg’s tests with eyes closed, statistically significant differences between injured and healthy legs in eight of them were observed. We believe that as these are more difficult tests, the balance system is pushed to a borderline situation and, therefore, the proprioception deficit presented by participants shows up more easily. By taking vision away and hindering the motor response, this leads to proprioception being forced to perform well.

A prediction for injured or healthy ankles of 65% with a 95% confidence interval using the variable “EC evaluation” was obtained. It was not found in the literature that this kind of analysis had been performed during an evaluation of the state of proprioception after ankle sprain. Because this variable was one of the 13 significantly different, and represented the other 11 for eyes closed, that appeared significant, the authors think this could be a good predictor variable. In the authors’ opinion, though the sample was not very large, the variable “EC evaluation” enabled classifying ankles as injured and healthy. This finding strengthens the initial idea that eyes closed tests should enable better detection of a proprioception deficit. However, the eyes open variable “AP movement in MREO”, also presented a predictive value of 64.5%, similar to that reported for “EC evaluation”. Nonetheless, it was decided that “AP movement in MREO” was of greater interest as a predictor variable, because it was obtained from the simplest test to perform. In fact, all subjects managed to perform this full test, both in healthy and injured group.

This study has certain limitations, as the homogeneity of the sample. The fact that the balance system deteriorates with age means that very elderly people in the population have very different absolute values. This limitation may have been partially covered with the use of relative evaluation variables. Furthermore, by having to use relative values, it would have been desirable to be able to use a healthy population database with Romberg’s eyes closed tests, but such a database was not found. Furthermore, no muscles that may be involved in ankle stability have been evaluated, as proximal muscles such as the gluteus medius (Kazemi et al., 2017), or peroneal muscles whose cross sectional area and thickness may be alterered in subjects with prior ankle sprains (Lobo et al., 2016). Other variables like, for example, myofascial trigger points, have not been assessed and may be associated with ankle pain (Sanz et al., 2016).

To conclude, measurements related to CoP after two weeks were altered in participants with ankle sprain. The fact that the ankle behaved almost normally under simpler conditions (MREO and MREO30mm) and, unprofitably, faced with more complex conditions (MREC and MREC30mm), could favour the individual apparently feeling well and returning to their usual work. In compromised situations the ankle could not respond correctly, favouring the onset of recurrent sprains that could lead to functional, and in some cases, chronic ankle instability, appearing in addition to osteochondritis and ankle arthrosis (Eils & Rosenbaum, 2001; Coughlan & Caulfield, 2007). Tests made with eyes closed (including Romberg’s test on a 30 mm thick foam rubber) and antero-posterior values were the most relevant variables, as showed the lineal regression equations. These should be taken in consideration on clinical evaluation in patients suffering from ankle sprains.

##  Supplemental Information

10.7717/peerj.4163/supp-1 Supplemental Information 1 Raw data

10.7717/peerj.4163/supp-2 Supplemental Information 2 Strobe checklist

## Additional Information and Declarations

### Data Availability

The following information was supplied regarding data availability: The raw data has been uploaded as a Supplemental File.
